# Study of Febrile Seizure among Hospitalized Children of a Tertiary Centre of Nepal: A Descriptive Cross-sectional Study

**DOI:** 10.31729/jnma.6092

**Published:** 2021-06-30

**Authors:** Ram Prasad Pokhrel, Radha Bhurtel, Kalpana Karmacharya Malla, Love Kumar Shah

**Affiliations:** 1 Department of Paediatrics, College of Medical Sciences Teaching Hospital, Bharatpur, Chitwan, Nepal; 2 Department of Nursing, College of Medical Sciences Teaching Hospital, Bharatpur, Chitwan, Nepal; 3 Department of pediatrics, Janaki Medical College, Janakpur, Nepal

**Keywords:** *febrile seizure*, *fever*, *season*, *status epilepticus*

## Abstract

**Introduction:**

Febrile seizure is the commonest cause of seizure in children and appears mostly between 6-60 months of life. The objective of this study is to find out the prevalence of febrile seizure among hospitalized children of a tertiary centre of Nepal.

**Methods:**

This is a descriptive cross-sectional study conducted in a teaching hospital of central Nepal, from 2014 January to 2019 December. After obtaining ethical clearance from Institutional Review Committee (Reference number: 2019-038), clinical and demographic data was retrieved from patient record retrospectively and reviewed for completeness and accuracy; those fulfilling the definition of febrile seizure were enrolled in the study. Convenience sampling technique was used. The data was analyzed using Statistical Packages for Social Sciences Version 25. Point estimate is done at 95% Confidence Interval and frequency and proportion was calculated.

**Results:**

Out of 4890 cases admitted during the study period, 214 (4.37%) (3.80%-4.94% at 95% Confidence Interval) children were diagnosed with febrile seizure. One hundred thirty one (62%) children had a simple febrile seizure. In majority of the cases, seizure lasted for less than 5 minutes; however, 10 (4.6%) of them presented with febrile status epilepticus, 111 (52%) children had generalised tonic seizure and upper respiratory tract infection was the commonest cause of fever.

**Conclusions:**

Prevalence of febrile seizure is significant among hospitalized children and simple febrile seizure is the commonest type. A substantial number of children present in febrile status epilepticus, even though the duration of febrile seizure is brief in most of the cases.

## INTRODUCTION

Febrile seizure (FS) is the most common form of childhood seizure occurring in 2-5% of children.^1^ However, its incidence has been found different in various parts of the world, 0.35% in China, 6.9% in Finland and 10% in India.^2-4^ These are more common in boys and the first episode usually occurs within first three years of life.^5,6^ It often occurs in the context of respiratory, gastrointestinal or urinary infection.^7^

Although FS are associated with viral infections, certain vaccinations, family history of febrile seizure or epilepsy,^8-10^ genetic and environmental factors also play a major role in the generation of this condition.^11^ Thus there might be variation in its prevalence and clinical characteristics from place to place.

This study aims to find the prevalence and clinico-demographic characteristics of febrile seizure.

## METHODS

A descriptive cross-sectional study was conducted at College of Medical Science Teaching Hospital (COMSTH) Nepal, from January 2014 to December 2019 after obtaining ethical clearance from Institutional Review committee (Reference number: 2019-038).

Children between the age of 6 to 60 months whose temperature was measured 38°C (100.4°F) or higher and had seizure which was not due to central nervous system infection or any metabolic imbalance, and that occurred in the absence of a history of prior afebrile seizures were included in the study.^1^ Fever was either reported by parents or recorded at the time of hospital admission. Lumbar Puncture and metabolic panel which included blood glucose, sodium, calcium and phosphorous were evaluated to exclude central nervous system infection and metabolic imbalance when clinically indicated.^1,12^ Simple and complex febrile seizures were categorized as per the definition given by Nelson and Ellenberg.^13^ Children having primary generalised seizures that lasted for less than 15 minutes and did not recur within 24 hours were categorized as simple febrile seizure (SFS). Conversely, children having seizures that were focal, prolonged (≥15 minutes), and/or recurrent within 24 hours were categorized as complex febrile seizure. Simple and complex FS are also referred to as typical and atypical FS respectively. Those having a convulsion or series of convulsions without gaining consciousness for 30 minutes or more were defined as Febrile Status Epilepticus (FSE).^14^

Sample size was calculated using the formula:

$$\begin{array}{ll} \text{n} & \text{= }{\text{Z}}^{\text{2}}\times \text{p}\times \text{(1-p)}/{\text{e}}^{\text{2}}\\ & \text{= }{\left(1.96\right)}^{\text{2}}\times 0.052\times 0.948/{(\text{0.01)}}^{\text{2}}\\ & \text{= }1894 \end{array}$$

Where,

n = Sample size
Z = 1.96 at 95% confidence interval (CI)
P = prevalence of febrile seizure from previous study, 5.2%^6^
e = margin of error, 1%

Data was collected by convenience sampling method and entered in the predesigned proforma which was entered in the excel sheet. Data was analyzed using Statistical Package for Social Sciences (SPSS) Version 25. Descriptive statistics which included frequency and percentage were derived and presented in tables and figures. Point estimate is done at 95% Confidence Interval and frequency and proportion was calculated.

## RESULTS

Out of 4890 cases admitted to the department of pediatrics during the study period, 214 (4.37%) (3.80-4.94 at 95% Confidence Interval) cases were diagnosed febrile seizure. There was no mortality due to FS. Most common age of presentation with FS was found to be 13 to 24 months. However, the first episode of FS occurred at the median age of 18 months. It was more common in boys with male to female ratio of 1.6:1 (Table 1).

**Table 1:** Demographic and clinical characteristics of children presenting with febrile seizure (n= 214).

Gender	n (%)
Male	134 (63)
Female	80 (37)
Age of Presentation (months)	Median=22 (IQR:36-15=21)
≤12	42 (20)
13-24	97 (45)
25-36	38 (18)
37-48	24 (11)
49-60	13 (6)
**Age at first episode of FS (months)**	Median=18 (IQR:30-12=18)
**Type of FS**	
Simple	131 (62)
Complex	83 (38)
**Episode of FS**	
First episode	152 (79)
Recurrent episode	62 (21)
**Temperature**	R=(105° F-100.5 °F) Mean ± SD=(101.9 °F±1.1)
Time between fever and seizure (hour)	
< 1	32 (15)
1-24	148 (69)
>24	34 (16)
Frequency of seizure	R=(7-1) Mean ± SD=(1.4 ± 0.9)
1 episode	157 (73)
>1 episode	57 (27)
Duration of seizure (minute)	
≤1	48 (22)
2-5	119 (56)
6-15	28 (13)
>15	19 (9)

**Figure 1:**
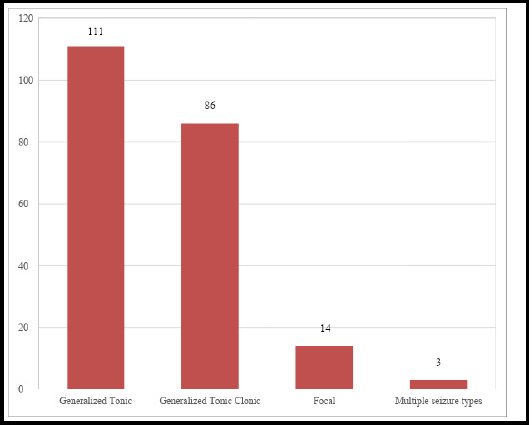
Types of seizure in children presenting with febrile seizure.

Temperature was measured between 100.5°F to 105°F with a mean of 101.9°F. Majority of the children had seizures within 24 hours of fever onset. Simple febrile seizures 131 (61%) were common compared to complex type 83 (39%). Among cases with complex febrile seizure, ten children presented with febrile status epilepticus, seven of which were experiencing the first episode of FS. In the majority of children, seizure aborted within five minutes. Children had 1 to 7 seizures during the current admission averaging 1.4 episodes per patient. The types of seizure encountered are illustrated in the (Figure 1) and the commonest was generalised tonic seizure 111 (52%) followed by generalised tonic-clonic seizure 86 (40%) and focal seizure 14 (6.5%). Three (1.5%) children had both generalised tonic as well as focal seizures.

**Figure 2:**
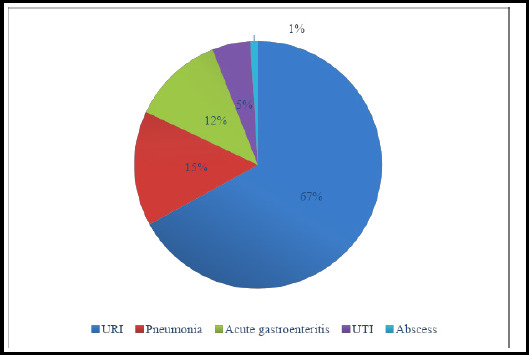
Causes of fever in children presenting with febrile seizure (n = 214).

Most common cause of fever in these children was upper respiratory infection followed by pneumonia as shown in (Figure 2). Cases of febrile seizure presented throughout the year in uniform manner which is shown in (Table 2). Among these patients, 11 (5%) had history of NICU admission, 13 (6%) had family history of epilepsy, 13 (6%) had family history of febrile seizure in first degree relatives.

**Table 2:** Seasonal distribution of cases presenting with febrile seizure (n = 214).

Seasons	n (%)
Summer (Mid May-Mid July)	39 (18)
Monsoon (Mid July-Mid September)	34 (16)
Autumn (Mid September-Mid November)	30 (14)
Early Winter (Mid November-Mid January)	38 (18)
Late Winter (Mid January-Mid March)	42 (20)
Spring (Mid March-Mid May)	31 (14)

## DISCUSSION

The present study found the prevalence of FS to be 4.37% which is comparable to other studies from Nepal and Turkey.^6,15^ From India, a higher prevalence of 10% and lower prevalence of 17.7/1000 has been reported prompting that even within a country the likelihood of having febrile seizure could differ.^2,16^ Age of presentation with febrile seizure in Pakistani children observed by Hussain et al^17^ was similar to Nepalese children as found in this study. Our study found that FS peaked during second year of life which corroborates to the finding from India and China.^18,19^ However Deng et al from Malaysia found its peak in the first year of life.^20^ In this study, FS predominantly occurred in male children similar to other studies from Nepal and abroad.^6,17^ It was more common in girls in Iran.^21^

Majority of the children (84%) had seizure within 24 hours of onset of fever which is consistent with the study from Pakistan.^17^ Simple FS was the commoner of the two types in this study as well as in others. ^6,21^ However, complex FS was predominant in studies done by Aggrawal et al from Nepal^22^, Al-Khathlan et al from Saudi Arabia ^23^ and Winkler et al from Tanzania.^24^ In this study, 4.6% of children with FS presented with febrile status epilepticus (FSE) congruent to the study from Ahamad et al.^25^ However 17.5% children from Saudi Arabia were reported to have FSE.^23^ We found that in most of the children, convulsions lasted for five minutes or less which is similar to the studies from Nepal and abroad.^6,26^ According to Deng et al from Malaysia, the children with FS had 1-9 episodes of seizure with mean of 1.5 episodes which is similar to the result from this study.^20^ While generalised tonic seizure was the most common type of seizure among children presenting with FS in this study, other studies found generalised tonic clonic seizure to be the commonest type.^6,27^

URI was the most common cause of fever in this study and others.^15,27^ Previous studies claimed the occurrence of FS mostly during winter and least likely in summer ^28,29^ unlike this study which found a similar number of cases through all seasons. In this study, Family history of FS in first degree relatives was present in 6% of children which is similar to the finding from a study in Dutch school children^30^ unlike a study from Hong Kong in which 18.2% of children had first degree relative with febrile seizure.^19^

Since most of the children have a single episode of FS, some might not reach hospital partly due to remoteness of their living place or their tendency to seek traditional healers rather than hospitals. Hence the true prevalence of the condition might not be reflected in the hospital based study like ours. The other limitation of this study could be the accuracy of data presented by parents regarding the duration and type of seizure, as their memory and judgment could be easily hampered by the anxious and fearful situation of witnessing their child having a seizure.

## CONCLUSIONS

Prevalence of febrile seizure is significant among hospitalized children. It was seen more commonly in boys. Its presentation peaks during the second year of life after which it gradually declines. Seizures occur mostly in the first one day after onset of fever and last less than five minutes, generalised tonic seizures being the most common type.
